# A Novel Biventricular Assist Device With a Single Driveline: A Report on the First Patient Treated

**DOI:** 10.1111/aor.70064

**Published:** 2026-01-06

**Authors:** Jan D. Schmitto, Jasmin S. Hanke, Sara Knigge, Zurab Darbaidze, Fanwu Kong, Torsten Heilmann, Alexander Weymann, Günes Dogan, Arjang Ruhparwar

**Affiliations:** ^1^ Department of Cardiac, Thoracic, Transplantation and Vascular Surgery Hannover Medical School Hannover Germany; ^2^ Shenzhen Core Medical Technology Co. Ltd Shenzhen China; ^3^ Avidal Group GmbH Bad Klosterlausnitz Germany

**Keywords:** BiVAD, biventricular heart failure, continuous flow VAD, heart failure, left ventricular assist device, LVAD, novel device, RVAD

## Abstract

We would like to report the first‐in‐human implantation of the novel DuoCor‐BiVAS biventricular assist device (BiVAD). This system features a single driveline and compact peripheral components, offering a promising mechanical support option for patients with terminal biventricular heart failure ineligible for heart transplantation.

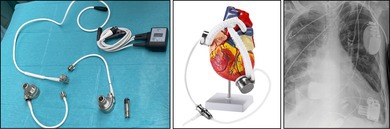

## 1 Introduction

**FIGURE 1:**
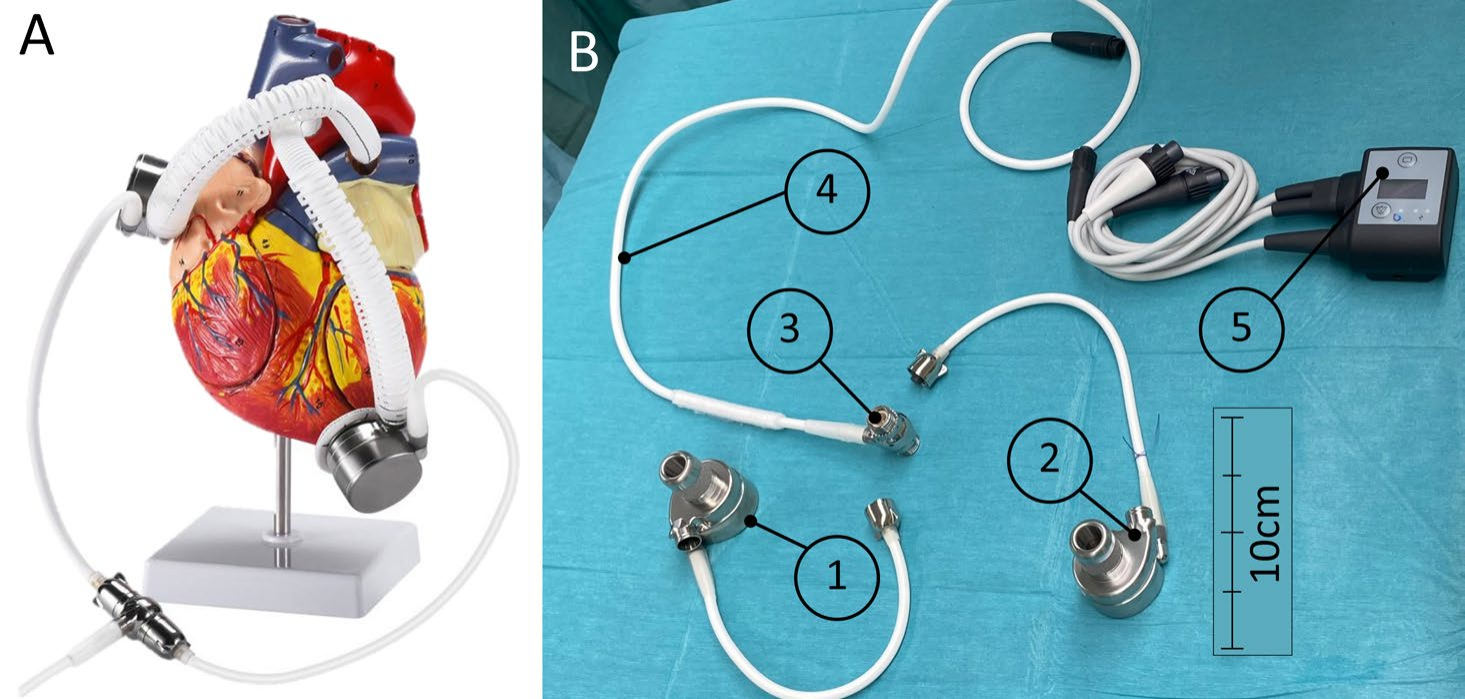
Anatomical model illustrating a heart with an implanted DuoCor‐BiVAS (A) and its internal and external peripheral components (B): left‐sided VAD component (1), right‐sided VAD component (2), driveline T‐connection (3), single driveline (4), and controller with pump connection and two battery connections (5). [Color figure can be viewed at wileyonlinelibrary.com]

**FIGURE 2:**
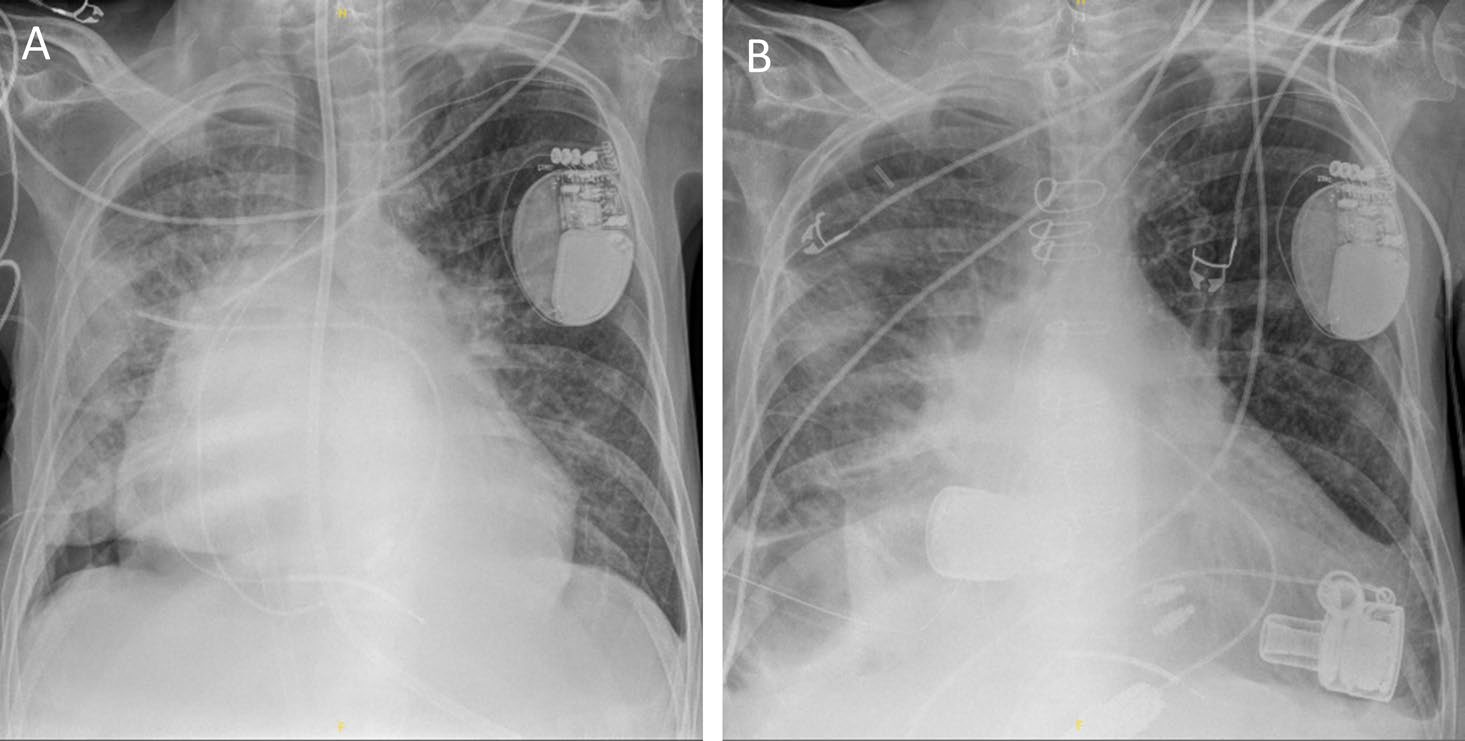
X‐ray images of the chest before (A) and after (B) the DuoCor‐BiVAS implantation. [Color figure can be viewed at wileyonlinelibrary.com]

The implantation of a biventricular assist device (BiVAD) is a rare and complex procedure often reserved for patients with terminal biventricular heart failure when heart transplantation (HTx) is not an option. Building on our experience with LVAD therapy [1, 2, 3], we present the first‐in‐human use of the novel DuoCor‐BiVAS system (Shenzhen Core Medical Technology Co. LTD, Shenzhen, China), which is characterized by a single driveline and compact peripheral components (see Figure 1B) [4]. The DuoCor‐BiVAS is approved by the Chinese authorities. The DuoCor‐BiVAS was previously tested in sheep over a 60‐day study period (Figure 2). The surgeon reviewed the data from this preclinical animal trial.

## 2 Case Report

In April 2024, a 60‐year‐old male patient underwent urgent implantation of the novel BiVAD DuoCor‐BiVAS due to terminal biventricular heart failure based on dilated cardiomyopathy and resulting cardiogenic shock. Due to multi‐organ failure as well as the urgency of the decompensation, the patient did not qualify for HTx. Assessment of right ventricular (RV) function was performed using both echocardiography and a Swan‐Ganz catheter. Echocardiography showed fulminant RV failure with an RV ejection fraction (RV‐EF) of less than 10% and a TAPSE of less than 10 mm, as well as an inability to wean the patient from ECMO. Preoperatively, after full cardiac decompensation—including 13 days on intensive care unit (ICU), 8 days of veno‐arterial ECMO support—and with the patient's consent, an interdisciplinary team decided to proceed with BiVAD implantation rather than attempting an intermediate step with a temporary device. The surgical approach involved a median sternotomy and bicaval cannulation for the heart‐lung machine. The left‐sided VAD component of the BiVAD was inserted into the left ventricle using a sewing ring, and the outflow graft was anastomosed to the ascending aorta. Similarly, the right‐sided VAD component was implanted into the right atrium, with its outflow graft attached to the pulmonary artery (Schematic illustration in Figure 1A). To prevent lung overfilling and thrombotic events due to low rotational speed, an iatrogenic outflow graft stenosis was created. Notable aspects of the surgical procedure included the utilization of silicon spacers to maintain precise pump alignment within the right atrium, which contributed to optimizing device placement and preventing suction events [5]. The driveline was tunneled to the right abdominal wall. Following the initiation of pump function, the heart‐lung machine was gradually weaned. Figure 2A and 2B shows an x‐ray of the chest before BiVAD implantation and BiVad implanted on right and left side of the heart.

The technical parameters for the left VAD were set at 5.38 L/min (3100 rpm), with a pulsatility index of 51, and a power consumption of 4.55 W. For the right‐sided VAD, the parameters were 4.17 L/min (3800 rpm), with a pulsatility index of 4, and a power consumption of 4.19 W. The lower flow and higher rotational speed of the right‐sided pump can be explained by the different designs of the right‐ and left‐sided pumps, which cause them to consume different amounts of power. In addition, various external factors, such as the connection of the graft to the pulmonary artery or aorta, different positions of the inflow cannula, and the patient's pressure conditions, could explain the higher rotational speed and lower flow observed in the right‐sided pump. The higher power consumption in the right‐sided pump is most likely caused by pneumonia‐induced RV afterload.

**FIGURE 3:**
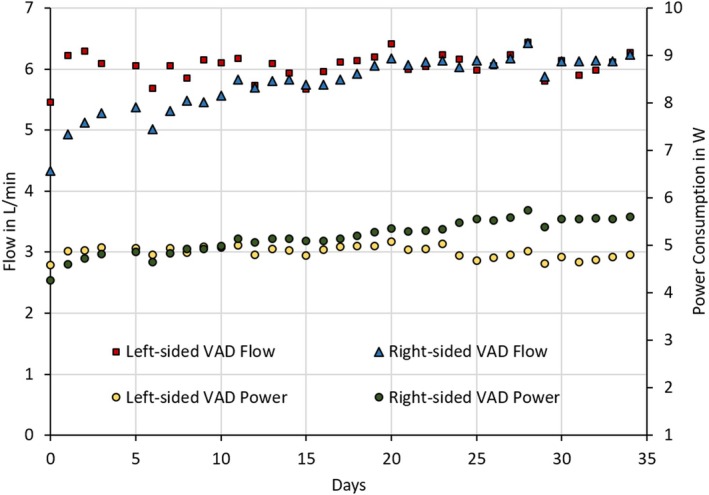
Daily summary of flow rates and power consumption for both the left and right‐sided pumps, recorded over a 35‐day period. [Color figure can be viewed at wileyonlinelibrary.com]

**FIGURE 4:**
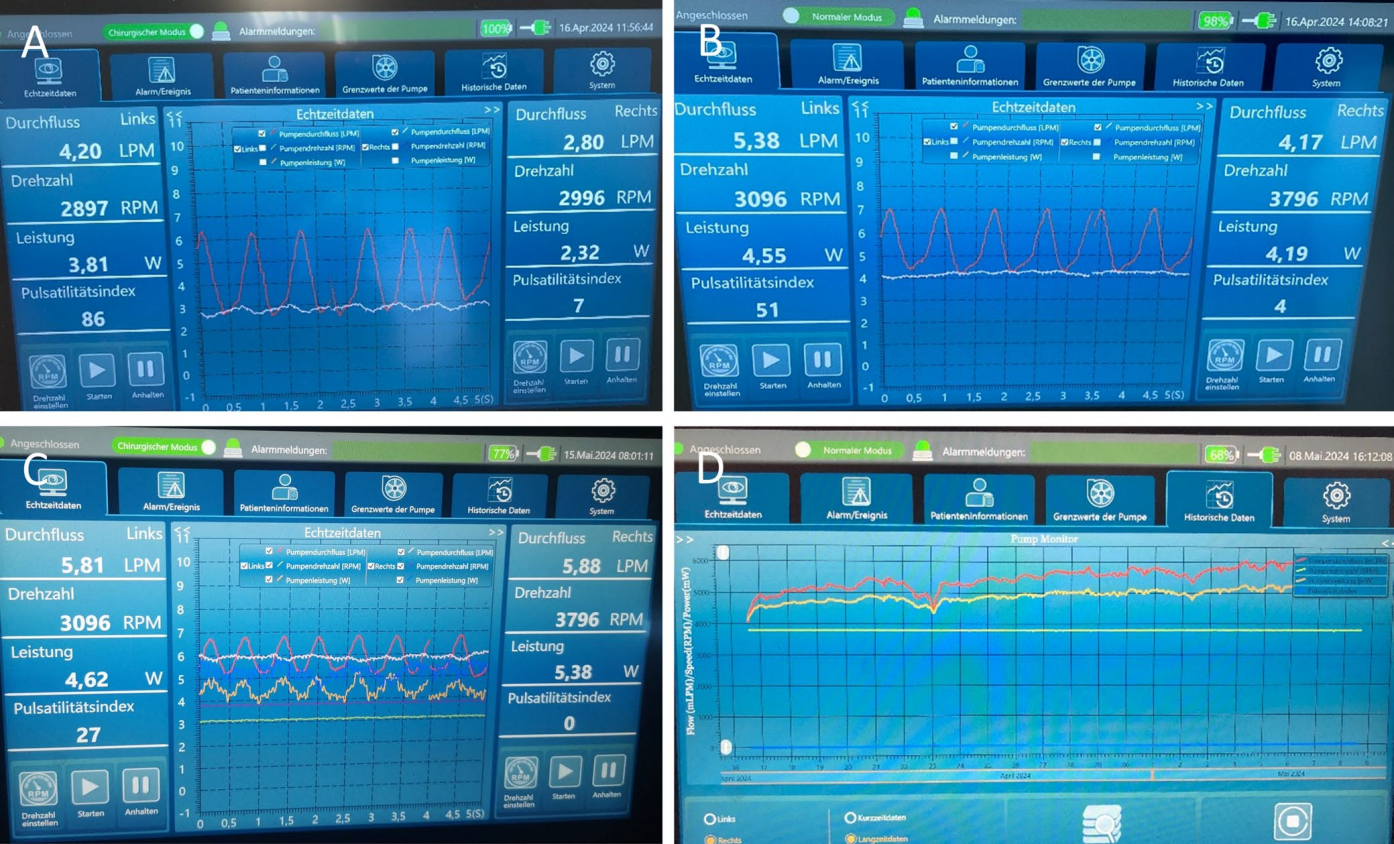
Monitor interface of the BiVAD system at different time points. (A) Intraoperative view during surgery; (B) monitoring in the intensive care unit (ICU); (C) bedside display 30 days post‐implantation; and (D) long‐term summary of internal measurements and parameters. (Translation note: “links” means “left” and “rechts” means “right” in German). [Color figure can be viewed at wileyonlinelibrary.com]

For the next 30 days postoperatively, the patient was hemodynamically stable, and the pump operated with constant parameters (see Figure 3). The INR was in the range of 2.0–2.5. No technical BiVAD malfunctions, pump thrombosis, bleeding, hemolysis, or stroke were observed. A CT scan was performed postoperatively and did not reveal any signs of pulmonary embolism. The system could be monitored and adjusted via an interface that enables both short‐term observation and long‐term recording of internal measurements and BiVAD parameters (see Figure 4A–D). The previously bedridden patient was successfully mobilized in the ICU and achieved walking distances of more than 60 m. However, after postoperative day 25 in the ICU, the patient developed pneumonia and sepsis. Neither the driveline nor other system components, nor the implantation itself, could be identified as the cause of the sepsis. Despite maximal intensive care efforts, the patient finally died 36 days after surgical implantation due to sepsis.

## 3 Discussion

The DuoCor‐BiVAS is a BiVAD consisting of two coupled magnetically levitated continuous‐flow pumps. These VAD components are connected within the thoracic cavity, allowing a single driveline to exit the body. As a result, only one external controller is required to manage both coupled pumps.

Compared to other treatment options—such as total artificial hearts (TAHs) or extracorporeal BiVADs like the Berlin Heart Excor—the DuoCor‐BiVAS offers several advantages. Its compact design is particularly suitable for smaller patients. In contrast, TAHs are often bulky and require significant anatomical space, necessitating preoperative compatibility assessments using 3D imaging (e.g., CT scans) [6].

While the Berlin Heart Excor is a well‐established option as a temporary option (bridge to transplant), especially in pediatric cases, it restricts patient mobility and poses challenges related to external cannulas, increased infection risk, device weight, noise, and psychological burden [7]. Especially, for patients with a low chance of transplantation a solution that can be transitioned into destination therapy is preferable.

Alternatively, the off‐label use of two continuous‐flow left ventricular assist devices (e.g., two HeartMate 3 pumps) results in the need for dual drivelines, batteries, and controllers—adding over 5 kg of equipment [8]. This setup can significantly impact patient comfort and quality of life when compared to systems utilizing a single driveline and control unit. In Table 1, the DuoCor BiVAS and the HeartMate 6 are compared.

**TABLE 1:** Comparison of the DuoCor‐BiVAS and the off‐label use of two HeartMate 3 in BiVAS configuration.

DuoCor‐BiVAS	Two HeartMate 3 in BiVAS configuration
One driveline	Two drivelines (increased the risk of driveline infection)
Around 14 h of battery	Around 16 h of battery
One controller	Two controller
0.8 kg system weight (including 2 batteries, 1 controller, one harness, implanted pumps, and driveline)	4.8 kg system weight (including 2 batteries, 1 controller, one harness, implanted pumps, and driveline)
Pumps are designed for the requirements of the different ventricles	Pump designed for left ventricle
Length inflow cannula left 20 mm	Length inflow cannula left 22 mm

In this compassionate‐use case, the DuoCor‐BiVAS provided stable and reliable hemodynamic support without adverse events, indicating its short‐term feasibility and efficacy in humans.

Due to regulatory and economic hurdles, the treatment gap for biventricular failure is increasing, particularly in Europe. The DuoCor device addresses this gap and therefore represents a significant innovation in the field of BiVADs. Further studies are needed to evaluate the DuoCor BiVAD and to investigate whether its technological features (e.g., reduced size, lower weight, a single driveline) will translate into improved outcomes for patients with heart failure.

## 4 Conclusion

The DuoCor‐BiVAS showed preliminary feasibility and effectiveness in its worldwide first human application, representing an innovative solution for treating patients with biventricular heart failure.

## Author Contributions

Jan D. Schmitto, Jasmin S. Hanke, Alexander Weymann, Günes Dogan, and Arjang Ruhparwar contributed to the conception and design of the case report and were involved in the clinical management of the patient. Jan D. Schmitto, Jasmin S. Hanke, Sara Knigge, Zurab Darbaidze, and Günes Dogan collected and interpreted the clinical data. Fanwu Kong contributed to device‐related technical aspects and data analysis. Torsten Heilmann provided engineering and technical expertise related to the biventricular assist device system. Sara Knigge and Zurab Darbaidze drafted the initial manuscript. All authors critically revised the manuscript for important intellectual content, approved the final version for publication, and agree to be accountable for all aspects of the work.

## Funding

The authors have nothing to report.

## Conflicts of Interest

The authors declare no conflicts of interest.

## Data Availability

The data that support the findings of this study are available from the corresponding author upon reasonable request.
